# Reconceiving orphan drug market exclusivity as a conditional public-law entitlement: a sustainable regulatory governance model with comparative lessons for China

**DOI:** 10.3389/fmed.2026.1896433

**Published:** 2026-07-22

**Authors:** Guiyu Quan, Yi Zhou, Yi Liu, Yinan Wu, Yong Yan

**Affiliations:** 1 School of Law, Sichuan Agricultural University, Ya'an, China; 2 School of Management, Sichuan Agricultural University, Chengdu, China

**Keywords:** access to medicines, conditional public-law entitlement, health equity, market exclusivity, orphan drugs, pharmaceutical regulation, public health policy, sustainable governance

## Abstract

**Background:**

Rare disease patients, who collectively number over 400 million worldwide, remain systematically underserved within existing universal health coverage frameworks. Orphan drug market exclusivity is a widely adopted regulatory incentive designed to stimulate pharmaceutical innovation for rare diseases. Recent regulatory reforms in China, the United States, and the European Union have introduced conditional mechanisms, signaling a shift from static proprietary protection toward a conditional regulatory governance model. Japan, while not undertaking legislative reform in 2026, offers a distinct reference point through its re-examination system. To date, no study has systematically integrated these latest legislative reforms into a comparative regulatory analysis or examined how the institutional design of orphan drug exclusivity can be calibrated to balance innovation incentives with equitable patient access.

**Methods:**

This study employs an integrated multi-method approach combining comparative legal analysis, doctrinal interpretation, evidence-based policy evaluation, and case analysis. A structured analytical framework is constructed around four key regulatory dimensions—eligibility criteria, exclusivity duration, scope of protection, and exception mechanisms. Using this framework, the study compares the orphan drug exclusivity regimes of the United States, the European Union, and Japan, and assesses the institutional risks embedded in China's newly enacted legislation.

**Results:**

This article proposes a novel theoretical model that reconceptualizes orphan drug market exclusivity as a conditional public-law entitlement whose legitimacy is premised on the continuing fulfillment of public health objectives. Applying this framework, the study identifies four core structural deficiencies in China's current regime and, drawing on mature comparative practices, develops targeted institutional design proposals for each regulatory dimension.

**Conclusions:**

Reconceiving orphan drug market exclusivity as a conditional public-law entitlement, rather than as an intellectual property right, offers a sustainable governance pathway for reconciling innovation incentives with the equity and financial protection goals of universal health coverage. Embedding conditional constraints into pharmaceutical exclusivity rules ensures that regulatory incentives serve long-term public health objectives and carries implications for addressing market failures and access challenges in other areas of global public health.

## 1 Introduction

The year 2026 marks a pivotal juncture for global orphan drug regulation and a defining moment for China's emerging market exclusivity regime. In January 2026, China formally introduced the market exclusivity provision for orphan drugs for the first time through Article 21 of the newly revised Implementing Regulations of the Drug Administration Law (1). The same month, the United States passed the Consolidated Appropriations Act, 2026, codifying into law the FDA's long-standing “indication-specific” interpretation of orphan drug exclusivity (2). The current Regulation (EC) No 141/2000 provides for a ten-year market exclusivity period, which remains the applicable law. Meanwhile, early in 2026 the European Union has reached political agreement on its long-awaited pharmaceutical reform package, which, upon its formal adoption and entry into force, will replace the current uniform 10-year exclusivity rule with a differentiated tiered protection system (3).

China's newly introduced orphan drug market exclusivity regime possesses several distinctive features that set it apart from the more mature frameworks of the United States, the European Union, and Japan. First, eligibility is determined by reference to a nationally administered rare disease catalog, rather than by a prevalence-based statutory definition or alternative economic criteria. Second, the exclusivity period is capped at a statutory maximum of seven years, and the marketing authorization holder is expressly required to commit to ensuring drug supply as a condition for enjoying this right. Third, the implementing details—including the specific eligibility criteria, the precise scope of exclusivity protection, and the various exception mechanisms—are largely delegated to subsequent departmental rules, which have yet to be formulated. The synchronicity of legislative adjustments across major jurisdictions also means that China's policy choice is no longer an isolated domestic legislative development, but is instead embedded in a broader international reflection on regulatory design. As rare diseases increasingly become a global public health priority, striking a proper balance between innovation incentives and drug accessibility is a critical issue bearing on health equity for millions of patients. It is therefore necessary to undertake a systematic comparison of these regulatory frameworks and to explore how an orphan drug exclusivity regime that is effective, equitable, and fiscally sustainable over the long term can be designed.

Most existing scholarship continues to characterize market exclusivity as a quasi-intellectual property right, treating it as a supplemental property right to patent protection that incentivizes R&D innovation and corrects market failure by conferring temporary market dominance (4). Yet this prevailing view has become increasingly difficult to reconcile with contemporary institutional developments. Recent regulatory reforms in China, the United States, and the European Union reveal that the legal logic underpinning orphan drug market exclusivity is undergoing a fundamental transformation.

This article argues that orphan drug market exclusivity should be reconceptualized as a conditional public-law entitlement rather than a quasi-intellectual property right. Within this theoretical framework, market exclusivity is not a vested reward automatically conferred upon drug approval, but a conditional legal status whose legitimacy depends on the continuing fulfillment of public health obligations. Its duration, scope of protection, and ongoing validity should therefore be subject to dynamic adjustment in response to evolving regulatory objectives and actual governance outcomes. This reconceptualization moves beyond the traditional innovation-centered paradigm by shifting the analytical focus from the conferral of rights to the imposition of accountability constraints, and from static protection to the ongoing justification of legitimacy.

As a latecomer in pharmaceutical regulation, China can fully learn from the regulatory practices of the United States and the European Union when establishing its brand-new orphan drug market exclusivity system. The orphan drug incentive mechanisms in both the US and the EU were developed against the backdrop of relatively mature pharmaceutical industries and medical reimbursement systems. In contrast, China's market exclusivity framework has only recently been implemented and faces unique practical constraints (5). An overly rigid exclusivity system that fails to fully address these constraints will simultaneously weaken pharmaceutical companies' incentives for innovation and equitable patient access to medicines. Through a systematic comparison of regulatory models in the EU, the US and Japan, this paper puts forward a refined and tailored institutional design for China: integrating affordability safeguards into the market exclusivity framework via conditional regulatory mechanisms.

## 2 Materials and methods

### 2.1 Comparative legal analysis

The core of this study lies in a structured comparative analysis of orphan drug market exclusivity regimes across the United States, the European Union, Japan and China. For the EU, the comparison encompasses both the currently applicable rules under Regulation (EC) No 141/2000 and the reform proposals set out in the 2026 pharmaceutical reform package, which has reached political agreement but awaits formal adoption and entry into force. These three jurisdictions were selected on account of their institutional maturity, representativeness, and the high degree of synchronicity between their 2026 reforms and China's recent legislative developments, which lends the comparison a unique policy window value, enabling an examination of the global evolution of orphan drug regimes from absolute exclusivity toward conditional exclusivity, and serving to corroborate the “conditional public-law entitlement” framework advanced in this article. The comparison is structured along four key dimensions—eligibility criteria, exclusivity duration, scope of protection, and exception mechanisms—and examines both the textual provisions and the operational logic of each regime, with the aim of generating actionable insights for China's institutional design.

### 2.2 Evidence-based policy evaluation

To assess the real-world effects of orphan drug exclusivity regimes, this study draws on empirical data from peer-reviewed literature, regulatory agency databases, including FDA, EMA, and PMDA orphan drug designation and approval records, and authoritative industry reports. These data are not deployed as standalone evidence but serve as empirical anchors that ground the legal analysis in documented policy outcomes, such as observed pricing patterns, generic entry delays, and patient access indicators.

### 2.3 Doctrinal interpretation of Chinese law

Article 21 of China's 2026 Implementing Regulations of the Drug Administration Law serves as the central legal text for normative analysis. Employing established interpretive methods, including textual interpretation, systematic interpretation that situates Article 21 within the broader regulatory framework, and purposive interpretation that examines legislative intent, this study identifies structural ambiguities, definitional gaps, and regulatory omissions within the provision.

### 2.4 Illustrative case examples

Representative judicial and administrative cases are examined to illustrate how abstract legal rules operate in practice and to identify institutional pitfalls that China should avoid. Key examples include the Catalyst Pharms., Inc. v. Becerra litigation, which triggered the 2026 U.S. statutory amendment clarifying the scope of orphan drug exclusivity, and the EU's Case C-237/22 on the adjudication of clinical superiority, which illuminates the operational complexities of exception mechanisms.

## 3 The theoretical foundations of market exclusivity: market failure, the trade-off between innovation incentives and access, and the sustainability imperative for reconceptualizing its legal nature

### 3.1 The legal concept of market exclusivity

The major jurisdictions define market exclusivity with differing emphases. However, in terms of a general summary of the common features of these regimes, the orphan drug market exclusivity period refers to a defined timeframe following the designation of a drug as an orphan product, during which the drug regulatory authority refrains from approving marketing applications submitted by other applicants for the ‘same' or a ‘similar' drug for the same indication, thereby conferring a de facto market monopoly on the original applicant. The core of this mechanism lies in furnishing an exclusivity incentive to drug developers through administrative inaction.

### 3.2 The incentive effect of orphan drug market exclusivity on pharmaceutical innovation: market failure and the necessity of incentives

#### 3.2.1 High costs, high risks, and long development cycles of orphan drug R&D and the scale of unmet medical need

Pharmaceutical research and development is inherently costly, yet the cost structure of orphan drug R&D exhibits distinctive features. The core difference between orphan and non-orphan drugs lies not in total R&D expenditure, but in per-patient cost and clinical trial architecture (6). A comparative study of drugs approved between 2010 and 2020 found that, although the overall clinical development costs of orphan drugs are typically lower, their per-patient R&D costs are substantially higher than those of non-orphan drugs (7). This represents a fundamental challenge inherent in conducting clinical studies within small, geographically dispersed patient populations (8). Furthermore, the development cycle for orphan drugs is considerably longer—rare disease drug development takes nearly four years more than non-rare disease drug development—and the clinical trial success rate for orphan drugs stands at approximately 6%. The combination of a low success rate and a prolonged development cycle further amplifies financial risk (9).

“Rare” refers not only to the small size of the affected population, but more fundamentally to the vast scale of unmet medical need. Although the prevalence of any single rare disease is extremely low, a systematic analysis based on Orphanet, the world's most comprehensive rare disease database, estimates the cumulative point prevalence of rare diseases at 3.5–5.9%, meaning that at any given point in time, approximately 263 to 446 million people worldwide are affected by a rare disease (10). China, with its enormous population base, faces a particularly severe challenge. As of 2025, there are already over 20 million rare disease patients in China, with more than 200,000 new cases reported each year (11), the public health impact of rare diseases continues to expand (12).

Furthermore, approximately 80% of rare diseases are genetic in origin, and the majority manifest in childhood, imposing severe physical, psychological, and economic burdens (13). The path to diagnosis is typically long and arduous: in the United Kingdom, the average time to diagnosis is 5.6 years, and 30% of affected children die before the age of five, with very limited progress compared to nearly a decade ago (14). In the United States, rare disease patients see more than ten specialists and experience three misdiagnoses on average before receiving an accurate diagnosis, with the entire process taking four to six years (15). Globally, only about 5% of rare diseases have an approved treatment, while approximately 95% remain without one (16), reflecting a structural deficiency in market-based resource allocation mechanisms.

#### 3.2.2 The necessity of market exclusivity as a regulatory incentive: empirical evidence from the United States

The U.S. Orphan Drug Act of 1983 serves as a highly instructive example, demonstrating that targeted regulatory incentives can effectively correct market failure in orphan drug R&D. The Act established a package of incentives, including a 25% tax credit on qualifying research expenses, exemption from FDA review fees, and—most critically—a seven-year market exclusivity period. In the decade prior to the Act, the FDA approved only about two rare disease drugs per year, and only 38 orphan drug products had reached the market before 1983 (17). Following the Act's enactment, the number of orphan drug designations more than quadrupled, rising from approximately 800 during the 1993–2002 period to roughly 3,600 during the 2013–2022 period (18).

**Figure 1:**
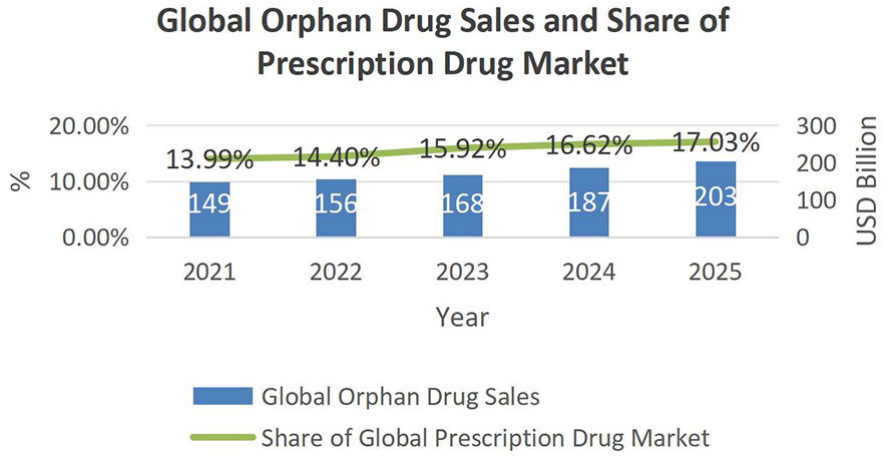
Global orphan drug sales and share of prescription drug market (2021–2025). Data source: Evaluate Pharma (63).

The success of the Act spurred global emulation: Japan enacted orphan drug legislation in 1993, followed by the European Union in 2000. Since 2000, the Committee for Orphan Medicinal Products has received 3,678 applications for orphan designation, issuing 2,399 positive opinions (19). Globally, the orphan drug market has expanded rapidly, growing from USD 188.45 billion in 2024 to an estimated USD 208.04 billion in 2025, representing a compound annual growth rate of 10.4% (20). Notably, 9% of approved drugs for orphan diseases subsequently achieved blockbuster status (21). Figure 1 further illustrates this growth trajectory, showing the sustained expansion of global orphan drug sales and their increasing share of the prescription drug market over the 2021–2025 period. The trend confirms that orphan drugs have become a commercially significant segment of the pharmaceutical industry, yet it also underscores the pressing need to ensure that market growth translates into improved patient access.

Among the various incentives embedded in orphan drug legislation, market exclusivity is widely regarded as one of the most significant. In its 2023 industry analysis, Biostock expressly noted that the Orphan Drug Act provides pharmaceutical companies that secure orphan drug designation with “three important financial incentives, the most significant of which is market exclusivity—erecting a barrier to direct competition for seven years following marketing approval” (22). In a systematic assessment of orphan drug incentives, Harris similarly concluded that market exclusivity has “effectively encouraged investment in orphan drugs” (23). It is thus evident that, in both industry practice and theoretical analysis, market exclusivity is regarded as one of the most central institutional instruments within the orphan drug incentive system.

### 3.3 Balancing innovation incentives and accessibility: how unconditional exclusivity creates barriers to affordable orphan drugs

Before examining the specific barriers that exclusivity can create, it is useful to locate market exclusivity within the broader regulatory pathway that determines whether a patient can ultimately access a medicine. This pathway involves at least four distinct stages: marketing authorization, the regulatory determination that a product meets quality, safety, and efficacy standards; market exclusivity, a period of protection against certain competing applications that operates after authorization; pricing and reimbursement decisions, typically made by bodies separate from the medicines regulator, which critically shape affordability; and actual patient access, the outcome at which all preceding stages converge. This article focuses primarily on the design of market exclusivity and its effects on the affordability and timeliness of competition. It should be emphasized, however, that even a well-designed exclusivity regime cannot by itself guarantee patient access; it must be complemented by appropriate pricing, reimbursement, and healthcare delivery mechanisms. The analysis that follows focuses on the impacts of market exclusivity's institutional design on patient medicine access.

#### 3.3.1 Exorbitant prices

Orphan drug prices are shaped by multiple structural factors, including small patient populations, high R&D costs, and limited therapeutic alternatives. Market exclusivity contributes to high prices primarily by delaying the entry of generics and biosimilars, enabling originator companies to sustain pricing in the absence of competitive pressure. The tension between the high pricing of orphan drugs and public budget constraints leaves the ethical obligation of “timely access” without workable boundaries in practice (24), and excessively high drug prices are consistently identified as the most significant access barrier (25). The difficulty in pricing orphan drugs lies not only in the price level itself, but in the absence of an evaluative framework capable of integrating multiple value dimensions (26). Empirical research further indicates that orphan drug pricing is shaped not merely by rarity, but by a confluence of factors, including the availability of alternative therapies and drug repurposing (27), and that its pricing should not be based solely on R&D costs or the monopoly position conferred by market exclusivity, but should instead incorporate a broader assessment of social value (26).

#### 3.3.2 Strategic exclusivity practices

One of the most widely criticized practices is “indication stacking,” whereby pharmaceutical companies obtain multiple orphan drug exclusivity periods for the same drug by securing approval for different indications within a broader disease category, thereby extending market protection well beyond the prescribed term and excluding competitors. Under such circumstances, companies can raise drug prices at will, undermining access (28). Another practice is the systematic label expansion from the rare disease market to the common disease market: some drug manufacturers first secure a high pricing anchor through a rare disease indication and subsequently achieve exponential sales growth by expanding into common disease indications (29). In addition, strategic behavior also takes the form of “subpopulation carving,” in which a pharmaceutical company artificially subdivides a common disease into multiple rare subpopulations, applying for orphan drug designation for each subgroup and thereby securing several independent market exclusivity periods (30).

#### 3.3.3 Delayed generic and biosimilar competition

Even after the orphan drug exclusivity period expires, competition frequently fails to materialize. A study examining orphan drugs in the Netherlands found that competition remained limited even many years after market exclusivity ended (31). Among 28 small-molecule orphan drugs, 39% had no generic registered; among 9 biologic orphan drugs, as many as 89% had no biosimilar. Even when generics did enter, this occurred at a median of 14.2 years—significantly longer than the 10-year exclusivity period itself. Moreover, even where competition did emerge, price reductions were modest, falling to 66% of the initial price, compared with 88% for products that faced no competition. This stands in sharp contrast to the non-orphan drug market, where generic entry typically drives prices down to 10–20% of pre-exclusivity levels within one to three years (32). Academic debates surrounding pricing and access have coalesced into three distinct narratives: one accusing pharmaceutical companies of excessive profit-seeking, another contending that high drug prices support innovation, and a third exploring alternative pricing models (33). These diverging perspectives reveal clearly that, regardless of one's normative stance, a scholarly consensus has emerged that the existing incentive and regulatory measures are inadequate in balancing innovation and access.

### 3.4 From quasi-intellectual property to conditional public-law entitlement: why the old theory no longer applies

In the early stages of orphan drug regulation, characterizing market exclusivity as a quasi-intellectual property right carried considerable explanatory force. Both mechanisms confer a legally protected exclusive position, incentivize innovation in areas where market incentives are inadequate, and create barriers to market entry. Where traditional intellectual property mechanisms were deemed economically insufficient as incentive instruments, market exclusivity functioned primarily as a regulatory substitute for patent protection. From this perspective, classifying orphan drug market exclusivity as a quasi-proprietary entitlement possessed intuitive plausibility and persuasive force at both the legal doctrinal and institutional functional levels (34).

However, as orphan drug regulatory frameworks have continued to evolve, the theoretical limitations of the quasi-intellectual property paradigm have become increasingly apparent. The most fundamental challenge to this framework arises from the application of profitability review mechanisms. Empirical evidence indicates that the orphan drug market is no longer composed solely of therapies targeting small patient populations with modest economic returns. This development undermines the traditional premise that orphan drug exclusivity uniformly protects innovations that are commercially non-viable (29). Under the EU orphan drug regime, the exclusivity period may be shortened if a drug becomes sufficiently profitable—a logic that departs fundamentally from traditional property rights theory. Patent protection, for instance, does not terminate prematurely merely because the patentee has earned an ‘adequate' economic return.

Drug supply obligations similarly expose this logical tension. Traditional intellectual property rights generally do not require the right holder to assume continuing supply obligations. However, the operational logic of orphan drug exclusivity has shifted toward being tied to supply assurance. Particularly when shortages jeopardize public health, exclusivity ceases to be a static exclusionary right and becomes instead an instrumental arrangement for realizing the right to health (35), a conditional regulatory entitlement premised on the continued alignment with public interest expectations, judicial divergences on this matter have also emerged in practice (36). Furthermore, features such as the clinical superiority exception and indication-specific limitations render the underlying architecture of orphan drug exclusivity increasingly difficult to reconcile with traditional quasi-property theory.

A new theoretical framework is therefore urgently needed to elucidate the underlying logic that dynamically balances innovation incentives, regulatory oversight, drug accessibility, and patient welfare imperatives. This study argues that market exclusivity should be defined as a conditional public-law entitlement. From this perspective, the legitimacy of market exclusivity flows not solely from its function as an innovation incentive, but more fundamentally from its institutional role in correcting market failure and advancing public health objectives; its continued validity must be premised on the ongoing fulfillment of these public goals. At the socio-ethical level, a discrete choice experiment conducted in the Netherlands found that approximately 36% of respondents supported special treatment for orphan drugs on the grounds of the right to health, 17% opposed it, and 46% varied their choices according to context (37). This finding lends support to our theoretical reconceptualization: the special status of orphan drugs is, in essence, a public-law arrangement grounded in social consensus, whose continuation is conditioned on public health objectives. Regulatory adjustments to the exclusivity period are intrinsic to the internal logic of the right itself, rather than constituting expropriation of, or improper interference with, a property right. Table 1 contrasts the two paradigms, highlighting the shift from a static property-based model to the conditional public-law entitlement framework proposed in this study.

**Table 1:** Paradigm shift in global orphan drug market exclusivity: from quasi-intellectual property right to conditional public-law entitlement.

Dimension	Traditional Quasi-IP paradigm	Conditional public-law entitlement paradigm
Source of legitimacy	Reward for innovation	Continuing alignment with public health objectives
Nature of right	Static exclusionary property right	Dynamic conditional regulatory status
Protection logic	One-time grant, fixed term	Ongoing conditionality, adjustable over time
Duration flexibility	Uniform fixed term	Differentiated; may be shortened or extended
Core constraints	Minimal continuing obligations	Supply assurance, profitability review, clinical superiority exception
Regulatory objective	Incentivize R&D	Sustainable balance between innovation incentives and equitable access

## 4 China's emerging regime: legislative evolution, normative structure, and sustainability risks

### 4.1 Legislative evolution and normative structure

Scholars have systematically mapped China's policy progress between 2012 and 2024 across areas such as orphan drug R&D incentives, accelerated review and approval, and the expansion of the national medical insurance formulary, noting that China has preliminarily established a policy framework spanning the full chain of “R&D–approval–supply–reimbursement” (38). The earliest signal of a market exclusivity period emerged in May 2022, when the NMPA issued the Implementing Regulations of the Drug Administration Law (Revised Draft for Public Comment), proposing for the first time a market exclusivity period of up to seven years for new orphan drugs (39).

Following extensive stakeholder consultation and policy refinement, the State Council issued the comprehensively revised Implementing Regulations of the Drug Administration Law on 27 January 2026. Article 21 establishes the market exclusivity period for orphan drugs and provides as follows: “For eligible orphan drugs, a market exclusivity period of not more than seven years shall be granted where the marketing authorization holder undertakes to guarantee drug supply. Where the marketing authorization holder fails to fulfill the supply guarantee undertaking, the market exclusivity period shall terminate. The specific conditions and measures for granting market exclusivity shall be formulated by the drug regulatory authority under the State Council.”

This legislative milestone marks the shift of market exclusivity in China from aspirational encouragement to a legally recognized exclusive right. However, Article 21 adopts a delegated provision model, vesting the authority to formulate specific rules in the NMPA. This creates uncertainty for pharmaceutical companies, which cannot reliably predict which drugs will qualify for exclusivity or what scope of protection they will receive. Delays in rulemaking may undermine the effectiveness of the incentive regime, and the absence of clear standards is likely to lead to inconsistent case-by-case determinations.

### 4.2 Legal risks across four key dimensions

#### 4.2.1 Narrow and rigid eligibility criteria based on disease catalogs

The most fundamental risk concerns the eligibility criteria for orphan drug designation. Article 21 limits eligibility to ‘eligible' orphan drugs, yet the criteria are not specified in the regulation, and China currently has no statutory definition of rare diseases; instead, rare diseases are identified through disease catalogs. The first two catalogs, issued in 2018 and 2023, cover a total of 207 diseases. However, as of 2025, approximately 1,400 distinct rare diseases have been identified in China, meaning the catalog coverage amounts to only about 15%. This implies that many serious, life-threatening diseases with no existing treatment—but not yet included in the catalog—cannot qualify for exclusivity incentives, potentially distorting R&D investment away from the most pressing unmet medical needs.

More critically, the effectiveness of the catalog-based approach depends heavily on the completeness of epidemiological data, which remains a significant weakness in China's rare disease governance. A study found that epidemiological data in China were available for only 62.81% of the 121 diseases in the first Rare Disease Catalog, substantially lower than the global figure of 92.56%. Even more critically, some diseases already included in the catalog have an actual incidence exceeding the prevalence threshold of 1/10,000 set by China's 2021 rare disease definition; for instance, the incidence of progressive muscular dystrophy reaches 2.53 per 10,000 (40). This suggests that the catalog criteria may diverge from epidemiological reality due to delays in data updating.

Furthermore, the term “orphan drug” itself requires interpretation. Does it cover only drugs approved specifically for a catalog-listed rare disease as their primary indication? How should drugs originally developed for common diseases but subsequently found to be effective for rare diseases be treated? The implementing rules must address these interpretive questions and provide predictable guidance for applicants.

#### 4.2.2 Inflexibility of the exclusivity period

A systematic review of four U.S. market exclusivity incentive legislations observes that each incentive regime critically lacks mechanisms to adjust for overcompensation, resulting in abuses and unintended consequences in every case (41), Article 21 of China's current regulation provides that the orphan drug exclusivity period shall be “not more than seven years,” suggesting that the duration may vary according to drug characteristics, but no specific adjustment mechanism has yet been introduced. This creates at least two types of risk. On the one hand, a uniform duration fails to account for differences across rare diseases in development timelines, clinical trial complexity, or manufacturing costs. Products with relatively low levels of innovation may receive the same seven-year protection as truly breakthrough therapies, potentially yielding windfall profits and diverting public health resources that could otherwise support more meaningful innovation. On the other hand, genuinely innovative therapies for ultra-rare diseases affecting extremely small patient populations may require longer protection to recoup development costs. The problem of insufficient innovation for ultra-rare diseases persists even in high-income countries (42), yet China currently provides no mechanism for extending the exclusivity period.

#### 4.2.3 Ambiguity in the scope of exclusivity

One scholar, focusing on the criteria for determining what constitutes the “same product,” has pointed out that a purely textual interpretation would limit “same product” to the biological and chemical identity of the drug's composition and structure, with therapeutic use falling outside its proper meaning (43). However, a systematic interpretation that takes into account Article 29 of the Draft for Public Comment—particularly the legislative purpose expressed in its first paragraph, which “encourages the development of new indications for rare diseases by already marketed drugs”—would suggest that therapeutic use should be incorporated within the scope of “same product.” This interpretive divergence illustrates precisely that, in the absence of a clear statutory definition, the seeds of institutional disputes are sown, generating substantial uncertainty for both originator and generic drug manufacturers. The U.S. Catalyst litigation illustrates just how costly and time-consuming such interpretive disputes can become (44). If China adopts ambiguous rules, it may well repeat the U.S. experience, becoming entangled in years of administrative and judicial disputes that not only raise compliance costs for companies but, more critically, delay patient access to medicines.

#### 4.2.4 Underdeveloped exceptions and flexibility mechanisms

A content analysis and bibliometric study of 61 national-level rare disease policy documents issued between 2009 and 2022 found that China's rare disease policy relies excessively on environmental-side and supply-side instruments, with a serious deficiency of demand-side instruments; within the category of environmental-side instruments, hard constraining mechanisms such as “regulatory control” account for a far smaller share than “strategic measures” (45). This dual imbalance indicates that, in designing the market exclusivity regime, policymakers have focused more on how to “confer rights” and “encourage R&D,” while paying insufficient attention to how to “constrain rights” and “safeguard patient interests.” This is the deeper reason why flexibility mechanisms such as the clinical superiority exception and profitability review have not been incorporated into the legislation.

It is commendable that Article 21 of China's Implementing Regulations already provides a “supply-failure-triggered-termination” circuit-breaker mechanism, linking exclusivity protection directly to continuous drug supply and embodying an institutional logic of “incentives conditioned on access.” However, China still lacks other exception mechanisms comparable to those in the US and EU, resulting in a potential lock-in effect: even a significantly superior new therapy cannot be approved within the seven-year exclusivity period, leaving patients stranded with suboptimal treatment for an extended time. Moreover, the regime also lacks the capacity to shorten the exclusivity period when a drug has become sufficiently profitable, as well as a public health exception that would permit accelerated approval of competing products during genuine shortages or public health emergencies.

## 5 Comparative analysis of market exclusivity across key regulatory dimensions

### 5.1 Defining eligibility for orphan drug designation

#### 5.1.1 United States

The Orphan Drug Act of 1983 defines a rare disease as one affecting fewer than 200,000 persons in the United States. The Act also provides an alternative economic criterion: a disease affecting more than 200,000 persons may still qualify for orphan drug designation if the costs of development cannot be recovered through sales. This dual-threshold system—primarily prevalence-based, supplemented by an economic criterion—is relatively permissive, enabling a broad range of diseases to secure orphan drug designation. Its permissiveness is further reflected in the equal incentives afforded to “partial orphan drugs”—drugs that hold both an orphan and a non-orphan indication. Data show that partial orphan drugs account for only 13% of all approved oncology drugs, yet contribute 35% of indication approvals and 30% of Medicare spending. Moreover, the median time from the first orphan indication to expansion into a common disease indication is only 1.3 years, and their monthly treatment cost is significantly higher than that of non-orphan drugs (46).

#### 5.1.2 European Union

In marked contrast to the permissive eligibility standards of the United States, the European Union adopts a more stringent, multi-criteria review model for orphan drug designation (47). To qualify for orphan designation, a product must simultaneously satisfy five core criteria: the target disease constitutes a distinct clinical entity, medical plausibility, seriousness, a prevalence not exceeding five in 10,000, and, where an authorized satisfactory therapy already exists, a demonstration that the proposed product offers “significant benefit.” Significant benefit functions as a gatekeeper mechanism at the designation stage, assessed by the Committee for Orphan Medicinal Products (COMP) during the orphan designation procedure. Its purpose is to ensure that orphan drug status is granted only to products that offer a clinically relevant advantage over existing satisfactory therapies already authorized for the same indication. It should be distinguished from clinical superiority, which involves a comparative efficacy assessment conducted in a different regulatory context. A clinically relevant advantage under the significant benefit criterion may be established through several pathways: inclusion in official treatment guidelines for the rare disease in question; the capacity to address unmet clinical needs in specific patient subpopulations that currently authorized medicines do not adequately cover; or the ability to replace existing therapies that carry serious long-term side-effect concerns. By channeling orphan drug incentives toward products that deliver meaningful therapeutic advances to patients, the significant benefit assessment operates as a pre-authorization screening mechanism that effectively targets regulatory rewards at genuinely innovative therapies.

#### 5.1.3 Japan

Japan defines orphan drugs as those targeting a disease affecting fewer than 50,000 patients domestically, where there exists high medical need and the drug demonstrates good development potential. In January 2025, Japan expanded its orphan drug designation system to permit designation based on patient subpopulations: even where the total patient population for a given disease exceeds 50,000, orphan drug designation may still be obtained if the patient subpopulation treatable with the specific drug numbers fewer than 50,000. Medical need is defined as the absence of alternative therapies or a demonstration that the new drug is superior to existing treatments; innovative drugs may also qualify for orphan designation even where existing treatments are available but are inadequate.

#### 5.1.4 Comparative Analysis

These three models reflect different value orientations: the United States pursues “maximum incentives” with a low threshold, but carries the hidden risk of companies achieving “evergreening of exclusivity” by subdividing disease categories; the European Union channels incentives toward products offering a clinically relevant advantage through its significant benefit assessment, a gatekeeper mechanism that screens out submissions unlikely to bring meaningful therapeutic advances to patients. Japan demonstrates flexibility through subpopulation designation, but insufficient rule transparency may lead to excessive administrative discretion. When designing its eligibility criteria, China must carefully balance its domestic industrial capacity, fiscal resources, and patient needs.

### 5.2 Duration of market exclusivity

#### 5.2.1 United States

An orphan drug receives a fixed seven-year market exclusivity period commencing from the date of FDA approval, regardless of the drug's level of innovativeness, development costs, or the size of the patient population. Research indicates that between 1985 and 2014, the proportion of orphan drugs whose exclusivity period outlasted their patent protection declined from 50% to 18%, suggesting that the same fixed term over-protects certain drugs while under-protecting others (48).

#### 5.2.2 European Union

Under the Regulation (EC) No 141/2000, which remains the applicable law, an orphan medicinal product enjoys a ten-year market exclusivity period from the date of marketing authorization. During this period, the European Union and its Member States shall not accept another application for a similar medicinal product for the same therapeutic indication, nor grant such an application or accept an application to extend the indication. Research suggests, however, that a uniform protection period may encourage firms to crowd into development around the same target. While some firms possess highly innovative mechanisms of action and face very low competitive pressure, other established firms operate in congested therapeutic areas with converging mechanisms of action and exhibit lower innovation indices (49). The 2026 EU pharmaceutical reform recalibrated orphan drug market exclusivity into a differentiated system precisely in response to this concern.

#### 5.2.3 Japan

Japan does not have a separate statutory market exclusivity period for orphan drugs; instead, de facto market exclusivity is achieved through its re-examination system (50). Following marketing approval, a new drug enters a re-examination period during which no generic applications may be submitted. For orphan drugs, the re-examination period can be extended to a maximum of 10 years. In practice, however, exclusivity protection is primarily driven by patents: 76% of orphan drugs have associated patents, 82% of which have obtained patent term extensions, exceeding the re-examination period by an average of 2.9 years (51).

#### 5.2.4 Comparative analysis

The U.S. model sacrifices institutional flexibility in exchange for a high degree of rule-based predictability, yet its fixed-term approach readily induces profit-seeking behavior on low-innovation drugs. The EU model forgoes administrative simplicity in favor of finely calibrated, differentiated innovation incentives, yet its tiered protection system tends to generate numerous legal disputes and designation controversies with blurred boundaries. Japan embeds exclusivity within its re-examination system; while stable in regulatory effect, this arrangement is also a significant factor contributing to delays in the market entry of new drugs. China should carefully weigh the strengths and weaknesses of these three models and seek a balance between certainty and flexibility.

### 5.3 Scope of exclusivity

#### 5.3.1 United States

The scope of orphan drug exclusivity has been the subject of extensive litigation and was ultimately resolved through a legislative amendment in 2026. Under the original statutory language, the exclusivity period barred the FDA from approving another application for the “same drug for the same disease or condition.” The FDA had long interpreted this as being limited to the specific approved “use or indication.” In Catalyst Pharms., Inc. v. Becerra, the Eleventh Circuit rejected the FDA's interpretation, holding that “same disease or condition” unambiguously referred to the designated rare disease itself, rather than a narrower indication within it (52). Section 6605 of the Consolidated Appropriations Act, 2026 replaced the phrase “same disease or condition” with “same approved use or indication within such rare disease or condition,” and the amendment was made retroactive. This legislative amendment codified the FDA's indication-specific approach, narrowing the scope of exclusivity to the specific approved use.

#### 5.3.2 European Union

Under the EU Orphan Medicinal Product Regulation, market exclusivity is granted on an indication-specific basis and applies to “similar medicinal products” for the same therapeutic indication within the orphan designation. The concept of “similar medicinal product” functions as a regulatory gatekeeping mechanism to determine whether a competing product falls within the scope of exclusivity. According to EMA/COMP practice, similarity is primarily assessed on the basis of the active substance identity. Where the active substance is identical, similarity is generally established. Where the active substance differs, a case-by-case assessment is conducted, taking into account molecular structure, mechanism of action, and relevant pharmacological characteristics. For biological medicinal products, structural attributes such as glycosylation patterns and post-translational modifications may be relevant, but they are considered within a broader functional and mechanistic assessment rather than as standalone criteria. EU orphan market exclusivity therefore does not constitute an absolute product-level monopoly, but rather a therapeutic indication-specific protection against the approval of similar medicinal products.

#### 5.3.3 Japan

Under Japan's re-examination system, the scope of exclusivity is not expressly delineated by statute but operates through the prohibition on submitting generic applications during the re-examination period. The scope of protection covers the approved drug and its approved indications; during the re-examination period, a generic drug cannot be approved for any indication. Because the system is not structured as an explicit grant of an “exclusivity period,” its scope boundaries are less formally defined than those in the United States or the European Union.

#### 5.3.4 Comparative analysis

The 2026 U.S. statutory amendment shifts to indication-specific protection as a means of curbing evergreening practices by pharmaceutical companies. The European Union, by adopting an indication-limited protection rule from the outset, avoided similar judicial disputes at the institutional design stage. Japan's re-examination system does not formally distinguish between different indications at the statutory level, yet in practice its exclusionary effect against generic market entry extends to all approved indications of the drug. This design of indication-limited protection rules serves a dual purpose and offers valuable reference points: on the one hand, it safeguards the originator's R&D returns for the specific rare disease indication; on the other, grounded in public interest, it permits subsequent manufacturers to develop and market alternative drugs for other indications within the same rare disease.

### 5.4 Exceptions and flexibility mechanisms

#### 5.4.1 United States

The FDA may approve a competing application earlier in the following circumstances:

1. The exclusivity approval has been withdrawn or the orphan drug designation has been revoked;
2. The holder's marketing application has been withdrawn;
3. The holder consents to another application;
4. The holder has failed to assure an adequate supply of the drug.

In addition, the Orphan Drug Act provides for a clinical superiority exception: where a follow-on drug constitutes the same drug for the same use or indication, the FDA will not recognize the follow-on drug's orphan drug exclusivity approval unless the applicant demonstrates clinical superiority. Clinical superiority may be established by demonstrating: greater efficacy; greater safety; a major contribution to patient care.

A notable example is Avadel's Lumryz, which, by virtue of its once-nightly dosing regimen, was found to offer a “major contribution to patient care” when compared with Jazz Pharmaceuticals' Xyrem and Xywav, each of which requires twice-nightly dosing, and was consequently approved during the exclusivity period on this ground.

#### 5.4.2 European Union

Under the Regulation (EC) No 141/2000, which remains the applicable law, Article 8(3) provides for three exceptions to market exclusivity:

1. The holder of the marketing authorization for the original orphan medicinal product has given his consent to the second applicant, or
2. The holder of the marketing authorization for the original orphan medicinal product is unable to supply sufficient quantities of the medicinal product, or
3. The second applicant can establish in the application that the second medicinal product, although similar to the orphan medicinal product already authorized, is safer, more effective or otherwise clinically superior.

In addition, the EU regime incorporates a profitability review mechanism: recognizing that orphan drug exclusivity incentives may generate excessive profits (53). At the end of the fifth year of market exclusivity, the 10-year period may be reduced to 6 years if it is established that the criteria for orphan designation laid down in Article 3 of the Regulation are no longer met, in particular where available evidence demonstrates that the product is sufficiently profitable such that the continued maintenance of market exclusivity is no longer justified. To initiate this procedure, a Member State shall inform the European Medicines Agency, which shall then commence the review. The marketing authorization holder is required to provide the Agency with the information necessary for this purpose. The 2026 pharmaceutical reform package, which reached political agreement in early 2026 but awaits formal adoption and entry into force, proposes to strengthen this mechanism by introducing additional performance-based conditions, including the possibility that unjustifiably high pricing may result in the loss of one full year of exclusivity protection. Until the reform enters into force, the current profitability review mechanism under Article 8(2) remains fully applicable.

#### 5.4.3 Japan

Japan's re-examination system provides less formal flexibility, containing no explicit clinical superiority or profitability review provisions. However, if safety or efficacy concerns emerge during the re-examination period, the Pharmaceuticals and Medical Devices Agency may take regulatory action, though this does not create a pathway for competitor entry.

#### 5.4.4 Comparative analysis

In terms of the comprehensiveness of exception mechanisms, the European Union has the most robust framework, followed by the United States, with Japan being the weakest. Too many exceptions risk undermining the innovation incentive value of exclusivity, whereas too few may leave patients dependent on suboptimal therapies for prolonged periods. China currently possesses only a single exception—supply failure—which is insufficient to address scenarios such as the market entry of superior new therapies. Accordingly, China should consider introducing more comprehensive exceptions and flexibility mechanisms when designing its regime. Table 2 consolidates the comparative findings across the four regulatory dimensions examined in this section, providing a structured overview of the orphan drug exclusivity regimes in the United States, the European Union, Japan, and China. It highlights the key similarities and divergences that inform the institutional design recommendations for China developed in the following section.

**Table 2:** Comparative analysis of orphan drug market exclusivity regimes across four key dimensions.

Regulatory dimension	United States (Current law)	European Union		Japan (Current law)	China (Current law)
		Current law	2026 agreed reform (Pending entry into force)		
Eligibility criteria	Prevalence < 200,000; alternative economic criterion; Subpopulation designation permitted	Prevalence ≤ 5/10,000; Disease seriousness; Significant benefit requirement	Unchanged	1. Prevalence < 50,000; 2. Subpopulation designation permitted; 3. High medical need	Catalog-based (207 diseases); no supplementary designation pathway
Exclusivity duration	Fixed 7 years	10 years	9 years (baseline); 11 years (breakthrough); 4 years (well-established use)	Re-examination period up to 10 years (not standalone exclusivity)	Maximum 7 years; no differentiation mechanism
Scope of exclusivity	Indication-specific (codified 2026)	Same therapeutic indication; Similar medicinal product	Unchanged	Covers all approved indications	“Same drug” undefined; scope not clarified
Exception mechanisms	1. Clinical superiority exception; 2. Supply failure exception; 3. Consent of the marketing authorization holder	1. Clinical superiority exception; 2. Supply failure exception; 3. Profitability review mechanism	Performance-based conditions added	No explicit statutory exception mechanisms	No clearly defined exception mechanisms

## 6 Toward a sustainable orphan drug exclusivity regime: refining China's institutional design

### 6.1 Refining the eligibility criteria for orphan drug designation

**Figure 2:**
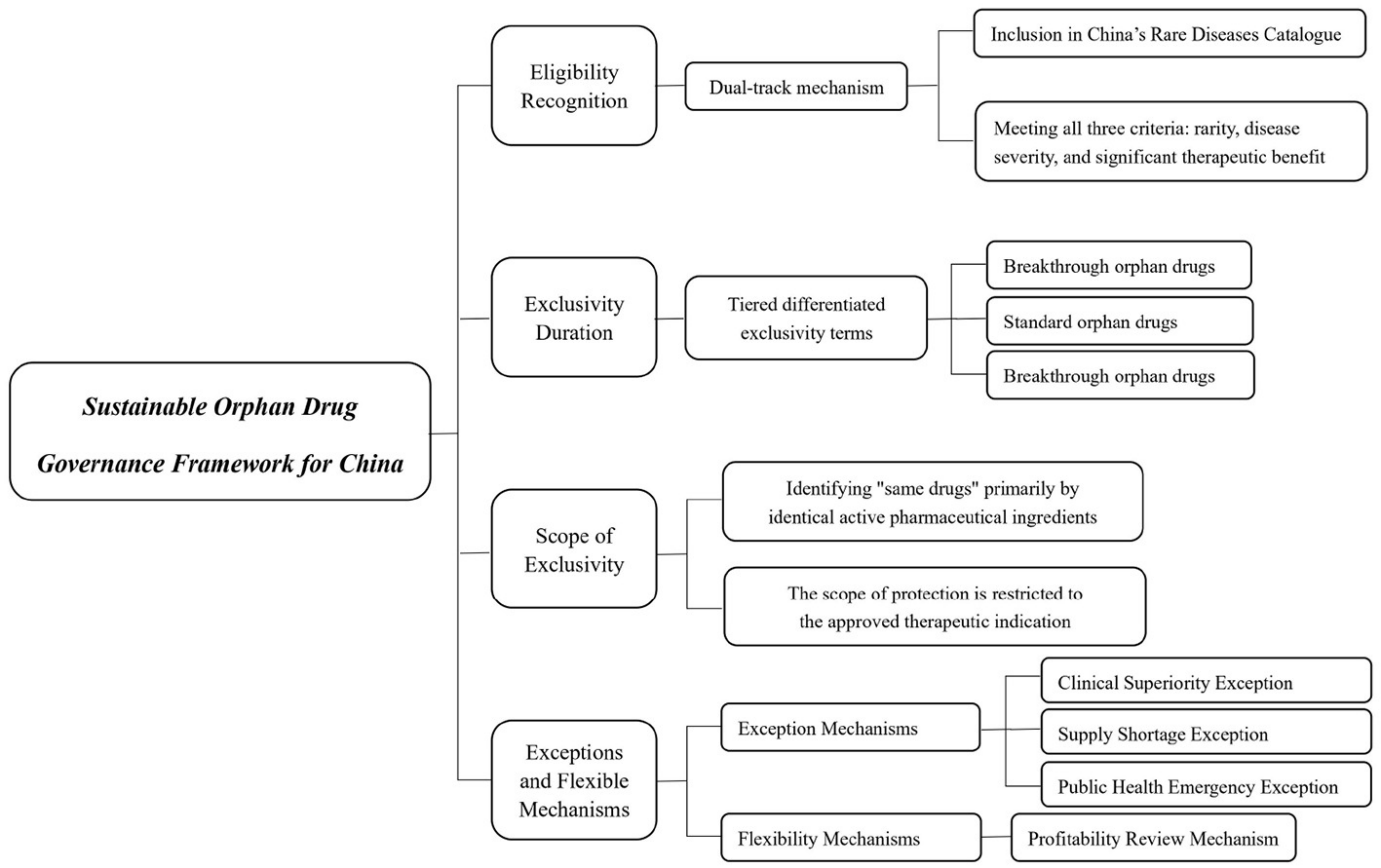
Proposed institutional design for sustainable orphan drug governance in China.

Figure 2 provides a schematic overview of the proposed institutional framework for China's orphan drug exclusivity regime. The framework is organized around four key dimensions—eligibility criteria, exclusivity duration, scope of protection, and exception mechanisms—and centers on a sustainable balancing mechanism that integrates innovation incentives, patient access, and fiscal responsibility. The following subsections elaborate on each component in detail.

#### 6.1.1 Addressing the limitations of the catalog-based approach

China cannot rely solely on a fixed rare disease catalog. Without the addition of scientifically sound supplementary designation pathways, rare diseases not yet included in the catalog may face prolonged incentive deficiencies, thereby delaying both drug development and treatment access for substantial numbers of rare disease patients. This study submits that China may draw upon the U.S. patient-population threshold model and the EU's provisions on life-threatening or chronically debilitating diseases together with its “significant benefit” standard to design a dual-track designation mechanism, under which a drug would be eligible for orphan drug designation if it satisfies either of the following conditions:

1. It treats a disease included in China's Rare Disease Catalogs;
2. The applicant demonstrates that the disease simultaneously satisfies the three criteria of rarity, seriousness, and significant benefit.

As to which authority would administer this mechanism, the NMPA is the appropriate body, given its existing statutory authority over drug registration under the Drug Administration Law. The NMPA could establish an orphan drug designation review procedure that parallels China's existing breakthrough therapy designation pathway, operational since 2020.

For Track 1, an applicant targeting a catalog-listed disease would be eligible for streamlined review, requiring primarily confirmation that the proposed indication matches the catalog entry. For Track 2, it should be noted that no globally uniform prevalence threshold for “rarity” currently exists. According to China's 2023 Rare Disease Definition Research Report, the research team lowered the prevalence threshold to 1/10,000. Academician Zhang Xue has also endorsed the view that defining rare diseases in China using a prevalence threshold of less than 1/10,000 may currently be the approach best suited to national conditions (54). The NMPA would evaluate applications with the support of an expert advisory committee convened from the Rare Disease Diagnosis and Treatment Collaborative Network. To prevent Track 2 from being misused for artificial subpopulation carving, the NMPA should require applicants to demonstrate that any claimed subpopulation constitutes a clinically distinct and medically justified entity—a safeguard that mirrors approaches developed by the FDA and the EMA.

The effective operation of the dual-track designation mechanism outlined above depends not only on rule design but also on the support of epidemiological and clinical data—data that are often severely lacking for rare diseases not yet included in the catalog. In this regard, China could fully leverage the data-support function of the Rare Disease Diagnosis and Treatment Collaborative Network. As of 2025, China's rare disease catalogs cover 207 diseases, and 419 hospitals participate in the collaborative network, engaging in case referral, remote diagnosis and treatment, and data sharing (55). This data infrastructure can provide an important basis for the epidemiological assessment of diseases not yet included in the catalog, thereby reducing the evidentiary burden on applicants. For diseases currently lacking sufficient data, a data accumulation period of one to two years may be established, during which applicants may access partial incentives, with full exclusivity granted only after formal confirmation of eligibility. An expert evaluation mechanism could supplement empirical data where clinical features are consistent with a rare disease but epidemiological evidence remains limited.

#### 6.1.2 Clarifying the scope of “Orphan Drug”: encompassing repurposed drugs

The term “orphan drug” in Article 21 of China's 2026 Implementing Regulations requires further interpretation. A key question is whether orphan drug designation is limited to drugs whose primary approved indication is a rare disease, or whether it also encompasses drugs originally developed for common diseases that are subsequently found to be effective for rare diseases.

The answer is clear: repurposed drugs are fully eligible for orphan drug designation. Under 21 CFR § 316.20, an applicant may request orphan drug designation for an “already approved” drug provided that the request is for a new orphan indication. Both the FDA and the EU permit such designation for repurposed drugs. For example, thalidomide was originally developed as a sedative and subsequently received orphan drug designation and approval for multiple myeloma and erythema nodosum leprosum. China should expressly include repurposed drugs within the scope of orphan drug eligibility, as such drugs typically come with a substantial body of existing safety data and a shorter development timeline, enabling them to deliver urgently needed therapies to the clinic at a fraction of the social cost of entirely novel drugs.

### 6.2 A Differentiated market exclusivity duration centered on the innovation–access balance

Within the theoretical framework of conditional public-law entitlement, the normative legitimacy of a uniform exclusivity period is subject to an even stronger challenge. Since the legitimacy of market exclusivity derives from its alignment with public health objectives, the duration of protection should be differentiated according to therapeutic value and the level of unmet medical need. From a legal doctrinal standpoint, the current Article 21 merely prescribes a maximum protection period of seven years and does not mandate a uniform fixed term for all drugs. The NMPA is lawfully vested with the authority to set differentiated durations of exclusivity protection in accordance with the specific conditions to be subsequently articulated.

Drawing on the differentiated exclusivity framework set out in the EU's agreed 2026 reform proposal, China could adopt a tiered approach that varies the protection period according to the degree of innovation and the level of unmet medical need. The proposed tiered structure comprises three levels:

1. Breakthrough orphan drugs: These would receive the maximum exclusivity period of seven years, reflecting their high therapeutic value and the substantial R&D investment required to bring them to market.
2. Standard orphan drugs: These would receive a baseline exclusivity period below the seven-year ceiling, preserving meaningful innovation incentives while avoiding uniform protection for products of differing therapeutic significance.
3. Repurposed or reformulated orphan drugs: For drugs that are repurposed for new uses or involve only minor formulation adjustments without significant clinical advantage, the exclusivity period would be set at a duration shorter than the standard baseline, based on factors such as clinical value assessment, anticipated generic competition, and drug pricing levels in China.

### 6.3 Further refining the scope of exclusivity

#### 6.3.1 Defining “Same Drug”

China should provide a clear statutory definition of “same drug” within its orphan drug market exclusivity regime. From a normative perspective, ambiguous rules for determining drug identity generate considerable legal uncertainty. If the boundaries of market exclusivity remain insufficiently defined, pharmaceutical companies may exploit minor formulation differences to extend their market dominance beyond the period that is properly commensurate with their actual innovative contribution. Accordingly, the criteria for determining a “same drug” should center on active ingredient identity and therapeutic equivalence, rather than on superficial differences in pharmaceutical form.

#### 6.3.2 Indication-specific protection

China should confine orphan drug market exclusivity to the approved rare disease indication and must not extend the scope of protection to the entire disease category. The legitimacy of market exclusivity derives from the drug developer's innovative contribution to a specific unmet medical need, not from an undifferentiated control over a disease area. To implement this principle, China should define “indication” by reference to the specific therapeutic use approved in the marketing authorization, including the disease or condition, the stage or severity of the disease, and the intended patient population. Where a drug is approved for a particular line of therapy, disease stage, or clinically distinct subpopulation, the exclusivity should attach only to that specific approved use, not to the broader disease.

Meanwhile, clear provisions should be formulated in China that a single drug cannot extend its total protection beyond a reasonable period by obtaining multiple independent exclusivity periods for different orphan indications. Specifically, if a drug is approved for a second orphan indication during the exclusivity period of the first indication, the exclusivity period for the second indication should run concurrently with the remaining term of the first exclusivity period, rather than adding a new, sequentially stacked exclusivity term. This rule prevents manufacturers from “stacking” exclusivity periods through sequential indication approvals.

### 6.4 Establishing exceptions and flexibility mechanisms to safeguard the innovation–access balance

#### 6.4.1 Clinical superiority exception

Establishing a clinical superiority exception is of paramount importance, if the market exclusivity regime obstructs patient access to a demonstrably superior treatment, the regime itself forfeits its legitimacy. Were it to preserve the market position of a first-to-market drug by barring the entry of a clinically superior product, the regime would subordinate patient welfare to corporate interests, contradicting the very public health rationale upon which it rests. Drawing on the combined experience of the United States and the European Union, China could adopt a flexible standard comparable to that of the EU when introducing a clinical superiority exception, such that satisfaction of any one of the three criteria—greater efficacy, greater safety, or a major contribution to patient care—would suffice:

1. Greater efficacy: The drug demonstrates a clinically meaningful improvement in efficacy over the approved orphan drug, established through head-to-head comparison in adequate and well-controlled clinical studies, with superiority demonstrated in clinically relevant endpoints such as overall survival, progression-free survival, or validated response rates.
2. Greater safety: The drug demonstrates a clinically meaningful improvement in safety over the approved orphan drug, established through comparative clinical data showing a substantial reduction in the incidence or severity of treatment-related adverse events, including serious adverse events or events leading to treatment discontinuation, or evidence of improved long-term tolerability in chronic use.
3. Major contribution to patient care: The drug offers a significant advantage in patient management that is not captured by efficacy or safety comparisons alone, such as a formulation or route of administration that enables treatment in settings where the existing therapy cannot feasibly be used, a pediatric formulation that addresses an unmet need in a specific patient population, or a dosing regimen that substantially reduces treatment burden and improves adherence. This advantage must be supported by evidence demonstrating its clinical relevance, such as pharmacokinetic data, patient-reported outcome measures, or implementation studies.

The burden of proof rests on the competing applicant, and the NMPA should make the determination based on the totality of evidence submitted, drawing on external expert advice where the assessment involves complex clinical judgments. If the competing party successfully demonstrates clinical superiority and obtains approval during the exclusivity period, the competing drug should be granted its own market exclusivity for the same indication, commencing from the date of its approval. The original exclusivity holder retains the exclusivity for its original drug, but the competing party may now market its clinically superior version. This dual-exclusivity outcome incentivizes genuine innovation while ensuring that patients receive better treatment.

#### 6.4.2 Supply shortage exception

Article 21 of the Implementing Regulations of the Drug Administration Law already makes the guarantee of drug supply a condition for the continuation of exclusivity; the supply shortage exception serves directly to reinforce and operationalize this condition. In the absence of such an exception mechanism, even if the holder repeatedly fails to supply the drug, patients would remain without access to an alternative during the exclusivity protection period. This would render the supply guarantee clause illusory and deprive it of any binding force.

Drawing on EU and U.S. practice, China could provide that where the existing orphan drug holder is unable to supply sufficient quantities to meet the needs of Chinese patients, the NMPA may approve a competing application during the exclusivity period. The following conditions must be satisfied:

1. The supply shortage is documented, for instance through hospital reports, patient registries, or the NMPA's own monitoring;
2. The holder has been afforded a reasonable opportunity to remedy the shortage;
3. The competing applicant is able to demonstrate its capacity to reliably supply the required quantities.

A reasonable period may be defined as 90 days, consistent with standard regulatory timelines for manufacturing remediation. The NMPA should maintain a public registry of documented supply shortages and the corresponding regulatory actions taken, ensuring transparency and predictability for both existing holders and potential competitors. This exception directly links exclusivity protection to the supply guarantee undertaking already required under Article 21. Failure to maintain adequate supply not only triggers termination of the exclusivity period but also permits immediate competitor entry under this exception.

#### 6.4.3 Public health emergency exception

A rigid market exclusivity regime that proves incapable of responding to major public health risks would stand in tension with the public-law foundation of drug regulation. The legitimacy of market exclusivity flows from its contribution to overarching public health objectives; when the continued maintenance of exclusivity protection would jeopardize patient welfare on a broader scale, the right should no longer subsist in absolute terms.

China should establish a public health emergency exception rule, providing that, in the event of a declared public health emergency, the NMPA may, upon the recommendation of the National Health Commission, grant temporary approval to competing products for the duration of the emergency. This exception would ensure that the exclusivity period does not obstruct access to life-saving treatment during a crisis. Such approvals should, however, be conditional and subject to post-market safety surveillance. With respect to the determination of a public health emergency, the NMPA should specify the types of evidence to be considered, such as a state of emergency declaration issued by the state.

#### 6.4.4 Flexibility mechanism: profitability review

Market exclusivity, as a form of “statutory monopoly,” tends to lead to excessively high orphan drug pricing, repeated acquisition of exclusivity periods, and the extraction of excessive profits by firms (56). When a drug has already generated commercial returns beyond a reasonable expectation, the continued maintenance of exclusivity protection ceases to be justified. In the absence of a review mechanism, the problem of excess monopoly profits will persist, and public skepticism toward “government-sanctioned monopoly” will continue to intensify. China could consider establishing a profitability review mechanism comparable to that of the European Union as an institutional basis for shortening the exclusivity period.

As to institutional responsibility, the review could be conducted by the NMPA, which holds regulatory authority over market exclusivity under the Drug Administration Law. Regarding the data required, the review should draw on at least two categories of information. The first is cumulative sales revenue of the orphan drug in the Chinese market since the grant of exclusivity. These data are obtainable from China's provincial drug procurement platforms, which record transaction volumes and prices for all publicly reimbursed medicines, and from the National Healthcare Security Administration's reimbursement claims database. The second is the R&D costs attributable to the orphan drug. In practice, such costs are often embedded within broader corporate financial structures and global development programmes, making precise attribution difficult. The NMPA could require the marketing authorization holder to submit a certified statement of China-specific development costs, subject to independent audit. Where cost data remain incomplete, the review could proceed on the basis of sales revenue alone, taking as a benchmark the revenue multiples observed in comparable rare disease indications where cost data are available. A phased approach, beginning with sales-based assessments and gradually incorporating cost data as methodological capacity develops, may offer the most feasible implementation pathway.

In practice, health technology assessment agencies have already established cost-effectiveness thresholds for rare diseases (57). However, such standards only apply to pricing and reimbursement procedures and are independent of the profitability review mechanism proposed in this paper. The profitability review is embedded within the market exclusivity regulatory framework. Even if the review concludes that the exclusivity period ought to be shortened, it will not automatically adjust the drug's pricing or reimbursement criteria. Instead, it serves as a regulatory signal indicating that material changes have taken place in the drug's market conditions.

Drawing on Japan's approach, China could attach post-marketing study requirements or staged approval conditions to breakthrough orphan drugs that receive extended exclusivity periods, so that the length of exclusivity is commensurate with the post-market obligations fulfilled by the holder. If the review concludes that the drug has achieved excessive returns, its remaining exclusivity period may be appropriately shortened, provided that a reasonable minimum protection period is preserved to ensure the enterprise still receives a basic return on its R&D investment.

## 7 Discussion

### 7.1 Research contributions and policy implications: revising the prevailing theory on the nature of market exclusivity and filling research gaps

Previous comparative law research has neither captured the latest 2026 regulatory reforms in the United States and the European Union, nor addressed China's newly enacted orphan drug exclusivity rules. By incorporating the most recent legislative revisions and adopting the analytical perspective of a late-mover jurisdiction, this study fills three major research gaps:

It updates the cross-jurisdictional comparative law evidence base beyond 2026;

1. It reconceptualizes orphan drug market exclusivity as a conditional public-law entitlement at the theoretical level, providing a novel theoretical lens through which to interpret emerging global regulatory trends;
2. It elucidates how emerging economies can draw lessons from the institutional shortcomings of mature jurisdictions and construct a more balanced regulatory framework from the top-level design stage onward.

For China, this study draws on comparative legal experience to propose a conditional market exclusivity regime, providing policymakers with a full-chain institutional framework spanning eligibility determination, tiered duration, scope of exclusivity, and exception mechanisms, thereby avoiding incentive failure or compromised access caused by regulatory gaps.

For emerging economies such as India and Brazil, this study illustrates how the late-mover advantage can be harnessed: drawing on the practical experience of mature jurisdictions, they can design a more balanced exclusivity regime from the outset and prioritize the development of regulatory capacity, thereby avoiding the “incentivize first, repair later” trajectory of their predecessors.

For mature jurisdictions such as the United States and the European Union, the comparative perspective adopted in this article provides a frame of reference for cross-regime assessment, enabling them to identify blind spots within their own systems. For instance, the United States could learn from the EU's differentiated exclusivity durations and profitability review mechanism, while the EU could draw on China's supply commitment condition.

Viewed from a broader perspective, the institutional exploration undertaken in this study holds relevance beyond the orphan drug context. Numerous emerging regulatory systems around the world face similar value conflicts in areas such as advanced biologics, gene therapies, antimicrobial resistance drugs, and pediatric medicines. When designing corresponding incentive regimes, the core insight of this study may serve as a reference: innovation incentive schemes should be designed as dynamic regulatory arrangements rather than as fixed, crystallized entitlements, so as to effectively stimulate pharmaceutical R&D while simultaneously safeguarding drug accessibility, thereby ensuring that the regime serves public health needs rather than industrial interests alone.

### 7.2 Research limitations

#### 7.2.1 Geographic limitations of the comparative law sample

The comparative analysis in this study focuses primarily on three developed jurisdictions—the United States, the European Union, and Japan—mainly because of their high institutional maturity and strong synchronicity with the 2026 reforms, which can provide a direct reference for China. In contrast, the regimes of some developing countries are still at the stage of policy exploration and lack the latest legislative developments commensurate with the 2026 reforms. However, the effectiveness of orphan drug policies is highly dependent on each country's level of economic development and healthcare financing capacity (58). The unique experiences of middle-income countries such as India and Brazil may offer greater reference value for China in certain dimensions. Due to space constraints, this study has not pursued this line of analysis. Future research may usefully undertake a dedicated examination of the comparative lessons to be drawn from orphan drug incentive regimes in developing countries.

#### 7.2.2 Insufficient analysis of the institutional environment and implementation capacity

This article focuses primarily on the normative design of the market exclusivity regime, while devoting insufficient attention to the institutional environment and implementation capacity upon which its operation depends. While the market exclusivity regime is undoubtedly an important supply-side incentive, regulatory approval does not directly translate into patient access (59). Its actual effectiveness in improving access depends heavily on complementary systems such as health insurance coverage, price regulation, and health technology assessment (60). China has shortcomings in areas such as clinical data infrastructure and domestic firms' capacity for original drug R&D, which may attenuate the actual impact of exclusivity incentives. Furthermore, the practice of Social Pharmaceutical Innovation demonstrates that market exclusivity, while an important incentive instrument, is not the sole pathway (61). Future research should focus on institutional landscapes in which multiple incentives operate in parallel.

#### 7.2.3 Operational limitations of the concept of “Drug Accessibility”

This study analyzes “drug accessibility” primarily through the two core variables of price and supply. However, accessibility itself is a multidimensional concept that also encompasses early diagnostic capacity, regional accessibility of medical services, and the extent of health insurance reimbursement coverage, among other factors (62). As a supply-side incentive instrument, market exclusivity cannot resolve the problems of “drugs without diagnosis” or “drugs without reimbursement.” Even a well-designed market exclusivity regime must be embedded within a sounder regulatory system to improve patients' real-world access to medicines. Further empirical research is required to evaluate how the institutional design of market exclusivity interacts with the aforementioned downstream policy factors to jointly shape drug access outcomes. Future studies may also conduct in-depth governance research covering other dimensions of orphan drug accessibility.

### 7.3 Directions for future research

Future research may proceed along four main avenues to further enrich the theoretical framework of sustainable orphan drug governance.

1. Expand the cross-national sample to include middle-income countries such as India, Brazil, and South Korea, and distill orphan drug policy lessons suited to developing-country contexts,
2. Conduct surveys, interviews, and collaborations involving confidential data to collect actual transaction prices and corporate profitability data, thereby enabling a precise assessment of the extent to which the exclusivity period aligns with the recoupment of R&D costs.
3. Conduct a longitudinal follow-up study five years after the implementation of China's detailed rules, systematically evaluating the long-term sustainability of the regime in terms of its impact on rare disease drug innovation, generic competition, and patient access.
4. Extend the theoretical reach of the framework by testing whether the conditional exclusivity framework constructed in this article can be generalized to other areas of market failure, such as drugs for antimicrobial resistance, neglected tropical diseases, and pediatric medicines.

## 8 Conclusion

This article reconceptualizes orphan drug market exclusivity as a conditional public-law entitlement, shifting the analytical perspective from a static intellectual property paradigm to a dynamic regulatory governance framework. The legitimacy of this entitlement rests on the right holder's continuous fulfillment of public health obligations.

China's newly enacted orphan drug exclusivity regime, set out in Article 21 of the 2026 revised Implementing Regulations of the Drug Administration Law, exhibits several distinctive institutional features: eligibility determined by a rare disease catalog encompassing 207 diseases, a statutory maximum exclusivity period of seven years conditioned on a drug supply guarantee commitment, and implementing details delegated to subsequent departmental rules that have yet to be formulated. Through a structured comparison across four regulatory dimensions—eligibility criteria, exclusivity duration, scope of protection, and exception mechanisms—this study identifies four categories of institutional risk: limited coverage of the rare disease catalog, an undifferentiated exclusivity term lacking tiered differentiation, an ambiguously defined scope of protection, and the absence of complementary exception mechanisms. Drawing on regulatory experience from the United States, the European Union, and Japan, this study proposes an institutional design built upon China's existing legal framework, constructing a calibrated regulatory model. It also offers a reference for other late-mover jurisdictions seeking to establish fair and resilient orphan drug governance systems.
